# Provider and user acceptability of intermittent screening and treatment for the control of malaria in pregnancy in Malawi

**DOI:** 10.1186/s12936-016-1627-5

**Published:** 2016-11-28

**Authors:** Deborah Almond, Mwayi Madanitsa, Victor Mwapasa, Linda Kalilani-Phiri, Jayne Webster, Feiko ter Kuile, Lucy Paintain

**Affiliations:** 1 Disease Control Department, London School of Hygiene and Tropical Medicine, London, UK; 2 College of Medicine, University of Malawi, Blantyre, Malawi; 3 Department of Clinical Sciences, Liverpool School of Tropical Medicine, Liverpool, UK

**Keywords:** Malaria in pregnancy, Intermittent screening and treatment, Acceptability

## Abstract

**Background:**

Malaria in pregnancy is a major cause of adverse maternal and fetal outcomes. Intermittent preventive treatment with sulfadoxine–pyrimethamine (IPTp-SP) is one of the control strategies promoted by WHO. In response to mounting resistance to SP, intermittent screening and treatment (ISTp) has been proposed as an alternative. The objective of this study was to explore the acceptability of ISTp for health workers and pregnant women.

**Methods:**

Semi-structured interviews of ten health workers and five focus group discussions of 38 women enrolled in an ongoing trial comparing IPTp-SP and ISTp with dihydroartemisinin–piperaquine (DP) were conducted at two antenatal clinics in rural Malawi. All transcripts were coded and themes were identified using a content analysis approach.

**Results:**

Amongst health workers, there were contrasting opinions on the preference of blood sampling methods, and the influence of method on reliability of diagnosis. The perceived greater effectiveness of DP compared to SP was appreciated, however concerns of user compliance with the full dose of DP in non-trial settings were raised. Despite the discomfort of repeated finger pricks, pregnant women were generally accepting of ISTp, particularly the chance for regular blood tests to check for infections and the perceived greater effectiveness with fewer side effects of DP compared to SP.

**Conclusion:**

In the trial context, pregnant women tended to prefer ISTp-DP over IPTp-SP. Health workers were also accepting of ISTp-DP as an alternative to IPTp-SP in light of increasing SP resistance. However, reliability of stock, adherence to malaria test results and user adherence to the full course of DP may present barriers to successful routine implementation. Effective communication with health workers and between health workers, pregnant women and their communities will be essential for the acceptability of focused antenatal care, including the best malaria control measures available.

## Background

*Plasmodium falciparum* infection in pregnancy has been associated with adverse clinical outcomes for both the mother and fetus, including severe maternal anaemia, low birth weight, pre-term delivery, and perinatal mortality [1]. Adverse effects of malaria in pregnancy can continue into early childhood, leading to anaemia [2–4] and an increased risk of malaria infection in the first and second years of life [5–8]. Women who live in rural areas, are in their first or second pregnancy [9], are infected with human immunodeficiency virus (HIV) [10], or are in adolescence, are at greater risk of infection [11, 12]. Pregnant women in moderate to high transmission areas often have asymptomatic infection that frequently remains undiagnosed and untreated [13].

The World Health Organization (WHO) recommends a package of interventions to control malaria in pregnancy [14], including the early diagnosis and treatment of malaria and the use of insecticide-treated nets (ITNs). In addition, intermittent preventive treatment (IPTp) is also recommended in the sub-Saharan Africa region. IPTp involves the administration of an efficacious anti-malarial drug at each scheduled antenatal care (ANC) visit in the second and third trimesters of pregnancy provided they are no less than a month apart, whether or not the woman is parasitaemic [15]. Currently sulfadoxine–pyrimethamine (SP) is the only anti-malarial used for IPTp due to its favourable safety profile [16], cost-effectiveness [17] and single dose regimen. Few anti-malarial drugs have a similarly long plasma half-life or can be tolerated as well during pregnancy, which has made an alternative to SP difficult to find [18]. However, increasing SP resistance is a clear concern for the efficacy and effectiveness of IPTp-SP [19, 20].

In the face of increasing SP resistance and lack of evidence on alternative drugs for IPTp, intermittent screening and treatment in pregnancy (ISTp) offers a possible alternative strategy for the control of malaria in pregnancy. The concept of ISTp is to provide scheduled screening for malaria using a rapid diagnostic test (RDT) and to treat RDT-positive women with a long-acting artemisinin combination therapy (ACT). The aim is to clear existing infections and provide additional post-treatment prophylaxis for 3–6 weeks. ISTp should be delivered at least three times during the second and third trimesters of pregnancy as part of focused ANC (fANC) [21] and ensures only those women who are parasitaemic are exposed to anti-malarial medication [22].

ISTp may be a promising alternative to IPTp-SP for the control of malaria in pregnancy [23]. However, its effectiveness under operational conditions depends in part on its acceptability to health workers and pregnant women, and opportunities for scale-up. The aim of this study was to assess the perceptions, experiences and attitudes of health workers and pregnant women towards IPTp-SP and ISTp using dihydroartemisinin–piperaquine (DP) within the context of a randomized controlled superiority trial (RCT) conducted in Southern Malawi [22].

## Methods

### Study site and context within the trial

Malaria is endemic to Malawi with an estimated four million episodes of clinical malaria annually [24]. Malaria infection during pregnancy remains largely asymptomatic with evidence of placental malaria in an estimated one in four deliveries [25]. According to the Malawi Malaria Indicator Survey (MIS) of 2014, 52% of the population had access to long-lasting, insecticide-treated nets (LLINs), with 70% of households owning at least one net. Within the same survey, 62% of pregnant women were reported to use a LLIN and 63% reported receiving two or more doses of IPTp-SP [24].

This acceptability study was conducted between June and July 2012 in Mpemba and Madziabango health centres in the Southern Region of Malawi, nested within a larger clinical trial of the efficacy of ISTp-DP compared to IPTp-SP. Methods of the main trial have been described elsewhere [22]. Briefly, HIV sero-negative women of all gravidae, between 16 and 28 weeks of gestation presenting for their first antenatal visit were enrolled and randomized to either the ISTp-SP or ISTp-DP arm. Women randomized to the control arm received SP by direct observation (DOT) at each antenatal visit, whilst those randomized to the ISTp-DP arm were first screened with a commercial RDT, and RDT-positive women were treated with a standard 3-day course of DP. The dose of DP was adjusted for the woman’s weight and all doses of DP were supervised. At enrolment, all women had blood samples taken by finger prick for HIV and haemoglobin assessment. At each antenatal visit, all women had a 5 ml venous blood sample drawn for assessment of study malaria endpoints and malaria screening in the ISTp arm, and routine care including screening for anaemia (at the last antenatal visit). RDT results were used to determine care. Malaria smears and PCR were used to assess the trial outcome of plasmodium infection during pregnancy; malaria smears were not used for point of care and read several weeks after they were taken. Live fetal endpoints were collected at delivery to assess babies born with a small weight for gestational age, low birth weight (<2500 g) or preterm (<37 weeks). *Postpartum* women and newborns were followed-up at 1 and 6 weeks after delivery. A LLIN was provided to each woman upon enrolment and all participation costs were met by the study.

Both trial staff and facility health workers involved in providing antenatal services at participating health facilities were interviewed. Only women enrolled in the trial who had completed the 6-week postnatal visit were recruited for the client focus group discussions (FGDs).

### Participant selection and interview procedures

#### Health worker semi-structured interviews

Health workers from Mpemba and Madziabango health centres involved in the delivery of antenatal services, and trial staff involved in the clinical trial were purposively selected to take part in the study. Trial research nurses were responsible for the majority of the trial duties. Health workers who were interviewed conducted ‘health talks’ to educate pregnant women about ANC and the trial. All eligible health workers (facility or trial staff) available on the days that the field team visited the health facility were interviewed.

#### Six-week *postpartum* women FGDs

Women who received either ISTp or IPTp-SP as part of the trial and were attending the clinic for their 6-week *postpartum* follow-up visit on the day of data collection were purposively selected to take part in the study [26]. As the recruitment for the FGDs depended on who was attending clinic for their postpartum follow-up on the day that the fieldworkers were at the facility, they were heterogeneous for trial arm to ensure they included enough people for discussion. This meant that some women in the same discussion group may have experienced IPTp-SP, while others may have experienced IST-DP; amongst those in the ISTp-DP arm, some women may never have had a positive RDT and so would not have received DP.

### Data collection

This observational study, embedded within an RCT, involved primary collection of qualitative data in the form of SSIs and FGDs. SSIs were administered to ANC health workers and trial staff and lasted between 45 min and 1.5 h. Interview topic guides included: health worker perceptions of malaria in pregnancy; experiences of the risk and consequences of malaria for pregnant women under their care; knowledge, experience and opinions of implementing ISTp as part of the trial; prior experience of conducting blood tests; and, opinions on the ISTp strategy if it were to be introduced as policy. SSIs were conducted in either English or Chichewa, or both in accordance to the preference of the interviewee. SSIs conducted in English were facilitated by DA; those conducted in Chichewa were facilitated by one of two trained fieldworkers fluent in both Chichewa and English.

FGDs were structured around a discussion topic guide to understand the experience and perceptions of women with respect to their recent pregnancy during the trial. Topics included: expectations of ANC visits; reasons for repeat visits; and experience with drugs, blood tests and, other services received during ANC visits. A key focus was to determine women’s opinions of repeated finger pricks and blood tests. Respondents were also asked to consider, based on their experiences, the potential acceptability of ISTp under operational conditions were it to become policy. Each FGD was carried out in Chichewa and moderated by two trained local facilitators.

### Data management

Each SSI and FGD was digitally recorded with informed consent, transcribed into Word and kept securely until analysis was completed. Those conducted in Chichewa were translated directly into English during transcription by the two local fieldworkers. All field notes were coded to maintain the confidentiality of participants. Translation and transcription was verified by a local social scientist for quality assurance.

### Data analysis

All transcripts were coded and themes were identified using a content analysis approach [26]. Information was coded using NVivo v9.2 and content analysis was based on inductive coding [26]. Themes were identified using a combination of a priori issues included in the discussion and interview guides that were informed by the research question, as well as emergent issues raised by the participants. Each participant and FGD was assigned a number, which is used in the presenting of quotes.

## Results

Ten health workers (all female) were interviewed, including five research nurses from the research trial team, and two nurse midwives, two nurse midwife technicians and one community nurse from the health facility staff. Seven were located at Mpemba Health Centre, two at Madziabango Health Centre and one divided her time between both facilities. Four FGDs of six to nine women each were conducted at Mpemba and one FGD of eight women at Madziabango. More FGDs were conducted at Mpemba due to the greater number of women recruited in the ISTp trial at this facility which resulted in a greater number of women attending the facility for *postpartum* visits during the period of this observational study. A total of 38 women took part in the FGDs; all were aged between 16 and 40 years old and most listed their occupation as ‘housewife’.

### Health workers

Five main themes were identified amongst health workers relating to the acceptability of ISTp-SP: blood tests, drugs, resources and stock, communication, and workload.

#### Blood tests

Health worker opinions on the acceptability of blood tests for ISTp consisted of concerns on the method of blood taking, the ability of the test to detect malaria, and rumours within the communities regarding blood taking.

##### Method of blood taking

Differing opinions were offered by health workers on their own perceptions and those of pregnant women on the alternative methods of blood sampling. In general, both trial and facility health workers perceived that pregnant women found blood tests uncomfortable irrespective of the method of sampling used, but did not believe this to be a barrier to repeat visits.

*“Some perceive the finger prick as painful; some perceive that peripheral [venous] is painful. We are told different things.”* (SSI 6, trial staff)

*“It is not a big deal, they feel uncomfortable but after the whole process is done they forget everything and are able to come again for the next antenatal clinic.”* (SSI 1, health facility staff)

A small number of health workers amongst both trial and facility staff felt that venous blood samples were more convenient owing to the multiplicity of tests that could be conducted on a larger blood sample derived from a single blood draw. However, the majority of interviewed health workers had a preference for finger prick tests for RDTs compared to venous samples due to finger pricks being simple and faster to administer. Another reported advantage was that smaller amounts of blood are needed for RDTs, which was more acceptable to pregnant women who worry about their blood “finishing”. The facility staff felt familiar with finger prick tests as they were also used for RDTs to detect HIV and syphilis during routine ANC.

*“I think RDTs are simple and it’s easier and even to the mother it’s acceptable… because they think the blood [taken venously] is too much, they say, ‘I’m pregnant and you are taking too much blood from me, how can I cope?’ but the RDTs they are more small and they don’t complain.”* (SSI 10, health facility staff)

##### Perception of RDTs and the influence of blood sampling procedure on the reliability of RDT results

One of the health facility midwifes interviewed reported that she did not trust RDTs because they sometimes gave negative results when clinical symptoms suggested malaria.

*“Testing with these RDTs, it may give negative test results yet clinically you are seeing that this patient has the signs typical of malaria. That’s when we say that I think these devices are not so effective.”* (SSI 7, health facility staff)

However, all other health workers interviewed (trial and facility staff) felt they could trust RDT results and that pregnant women were also confident in the diagnosis as they could see the results of the RDT. Some health workers expressed doubt over the reliability of microscopy due to human error and suggested that RDTs were more reliable, especially when health workers had less experience.

*“They don’t doubt when we are using the RDT process because they see the whole process, unlike when we are using the microscope process.”* (SSI 1, health facility staff)

*“It is effective because when one is positive it means she is indeed positive, unlike the microscope way because sometimes it’s difficult for the laboratory technician to detect the parasites and the results can come out negative when one is positive.”* (SS1, health facility staff)

*“I prefer RDTs because in the microscopy, you need experience.”* (SSI 9, health facility staff)

Comments made by a small number of health workers/trial staff revealed that the method of blood sampling can influence confidence in the reliability of RDT results; these health workers felt that venous blood samples detected ‘real’ malaria due to drawing directly from the vein.

*“Since we go straight into the vein to take the blood, I think we can determine that this is the real malaria in the stream.”* (SSI 2, health facility staff)

##### Community rumours regarding blood draws

Health workers reported that they were aware of rumours and misconceptions within the community regarding blood draws for screening tests in the trial, the most prominent belief being that the blood taken would be sold.

*“They come to the clinic and you draw blood maybe to do a certain test, they feel like you’re selling their blood.”* (SSI 6, trial staff)

Other beliefs reported were blood being used for reasons other than those explained in the trial and that blood would be finished in the mother, leaving inadequate blood for the baby. Health workers believed these rumours which arose during the trial would remain in the event that ISTp were to be introduced into routine service delivery. However, health workers felt these misconceptions were not necessarily a barrier to implementing ISTp, as evidenced by the continued compliance of women in attending scheduled antenatal visits during the trial where venous blood draws were routinely conducted; they believed that these rumours could be addressed by simple explanation to the women in a way they would understand of the convenience of venous blood draws for conducting several blood tests from a single sample. They also stressed the importance of informing and involving husbands and community leaders who have strong influence on pregnant women’s care seeking.

#### Drugs

Several opinions regarding drug effectiveness and preferences were expressed by the health workers regarding the decreased effectiveness of SP for prevention of malaria due to SP resistance, the relative effectiveness of SP and DP, prevention versus treatment and drug side effects.

##### Low effectiveness of SP to prevent malaria due to SP resistance

The majority of health workers felt an alternative drug to SP was required as SP was no longer effective in preventing pregnant women from malaria infection due to SP resistance. They also reported that the vast majority of women attending antenatal care services at the facilities no longer believed SP prevented malaria and regularly asked to enrol into the ISTp trial to benefit from DP which they believed was more effective in treating and preventing malaria.

*“They differentiate between SP and DHA [DP]. Most of the women want to be on ISTp because they know that it is a new drug that we are testing.”* (SSI 5, trial staff).

##### DP compared to SP

The overriding opinion of the facility health workers and trial staff interviewed was that DP was more effective than SP as exemplified by the infrequent unscheduled visits made by women who received DP.

*“If a woman is on ISTp and is taking DHA [DP], it’s rare that the woman might come to the clinic with malaria, unlike that on IPTp.”* (SSI 8, trial staff)

Health workers expressed approval of weight-based dosing of DP as opposed to a set dose for everyone, although some felt that outside a trial setting the correct dose would more likely be taken if only whole tablets were given due to time constraints in cutting tablets.

*“I know how hospitals work* – *we have lots of patients. If the government decide to go with this, are they going to manage giving three and a half tablets to someone? Will they have time maybe to give one half according to the protocol?”* (SSI 3, trial staff).

##### Prevention versus treatment

There was consensus amongst health workers that prevention is better than cure. However, with the wide perception of decreased efficacy of SP and a feeling that women prefer not to take drugs during pregnancy if they are not ill, many placed emphasis on the promotion of ITNs rather than IPTp-SP for prevention. It was also a common opinion that prevention and treatment should work “hand-in-hand” so that if prevention has failed, a woman would receive effective treatment.

*“If the woman is not given a bednet then this woman will be coming frequently to the hospital with malaria, so I think to prevent first and we can give treatment if prevention is failed”* (SSI 10, health facility staff)

##### Drug side effects and adherence to treatment

All health workers involved in the trial gave a favourable opinion of DP over SP owing to fewer side effects seen with DP.

*“There are few side effects [of DP], if any, I’ve never heard of severe side effects.”* (SSI 3, trial staff).

However, acceptance by pregnant women to adhere to a 3-day DP course without DOT was a clear concern by health workers. In the trial, women were provided with transportation reimbursements to enable them to attend supervised dose administration at the clinic, which would not be possible under routine service delivery. As such, health workers feared women would be unable to travel to ANC daily to receive DOT due to long distances, limited transportation and costs.

*“These women come from afar, so they just buy drugs from the shops without coming to the hospital.”* (SSI 8, trial staff)

Other concerns included the perception that if women vomited a dose of the drug at home, they would not be able to repeat the dose and that some women would not receive permission from their husbands to access treatment. Health workers used what they had heard women say about taking SP to form their opinions of how likely it is they would take DP.

*“They will just say, ‘I will take the medicine at home because I am coming from very far so I cannot take SP here, I have to take it home after eating food,’ but then when they go home they just drop the treatment without taking it.”* (SSI 8, trial staff)

There was wide agreement amongst trial and facility health workers that women in the trial would not have completed each course of DP if doses two and three had been allowed to be taken at home. However, it was also agreed that if ISTp were to become government policy, adherence to all doses being taken under supervision at the clinic would be high as it would be seen as compulsory.

*“If it was a government policy, they will be coming [to ANC to take DP].”* (SSI 5, trial staff)

#### Resources and stock

A key health worker concern was the likelihood of a continuous supply of RDTs and medication were ISTp to be introduced as policy. All of the non-trial health workers interviewed reported that their facility had experienced stock-outs of RDTs outside of the trial. Some maintained that SP is always in stock, while others said government hospitals routinely run short of drugs. However, they noted that the government has been supportive of the study and point to this as evidence of the government’s commitment to ISTp.

Although presence of stock is a feasibility issue regarding implementation of ISTp, it also has implications for health workers’ acceptance of the intervention, which appeared to be conditional on a reliable supply of RDTs, medicines and other supplies:

*“…If we adopt the ISTp then we are relying on RDT, then [if RDTs are out of stock] we are going back to IPTp* – *which is very confusing [for the pregnant women] … If we have the continuous supply of RDT then ISTp will be a success.”* (SSI 9, health facility staff)

#### Communication

Health workers were unanimous in saying there was good communication between both nurse and patient and between themselves and their colleagues. They felt ANC attendance was very good with women only occasionally missing their appointments, adding that they felt confident women would attend scheduled screening visits. The need for efficient communication and understanding between health workers and between health workers and their clients and communities was deemed essential in acceptance of ISTp by these groups.

*“… The problems are not that big that we cannot overcome them. We can explain to those people who are involved like the village head committee in assisting us in explaining to the patients and people in the village because it has to start in the community so that when they come here, they should not be surprised with what’s happening at the hospital. So just involving these people I think everything can go on well.”* (SSI 7, health facility staff)

#### Work load

Some health workers expressed concern that ISTp would increase their work load as there is “more to be done” compared to IPTp.

*“I think that there can be challenges in areas where workers are few. There can be difficulties with follow up of patients… and also just doing malaria tests.”* (SSI 7, health facility staff)

There were suggestions that ISTp could be made more acceptable to health workers as far as additional workload was concerned by incorporating the screening component into tasks already carried out at ANC with task shifting to lower cadre staff such as health surveillance assistants.

*“It can be done after they do the antenatal clinic at the booking; they train the HSAs [Health Surveillance Assistants] doing the RDTs for HIV and syphilis, so it can be done at the same time, with the HIV and syphilis test and the Hb, so it can be done.”* (SSI 4, trial staff)

### Trial participants

Three main themes were identified amongst pregnant women relating to the acceptability of ISTp-SP, relating to blood tests, drugs and reasons for repeat visits.

#### Blood tests

Several sub-themes around blood tests emerged that influenced the acceptability of ISTp with DP compared to IPTp-SP, including: information dissemination by health workers of the importance of blood tests; blood sampling methods; the relation of blood sampling methods to reliability of test results; the interpretation of the importance of blood tests; and social rumours about blood sampling.

##### Communication by health workers regarding blood tests

Communication between health providers and pregnant women was described as being good overall. Some women expressed disappointment that reasons for their blood tests were not explained to them by the facility health workers but this was not a shared opinion by the majority of the women. Most women believed blood tests to be very beneficial. When told the results, women reported to have felt more knowledgeable of their wellbeing and that of their unborn babies and empowered to ensure their continued health.

*“We saw this as a great and good thing to know how our body is functioning because we used to walk without knowing.”* (FGD 1)

##### Blood collection methods

The sentiments expressed by most women regarding blood sampling revolved around the pain that accompanied the sampling procedure. Opinions were however divided on which method was the more painful.

*“Finger pricks are painful. It’s much better from the vein.”* (FGD 5)

*“I think that finger pricks are good because it’s not like they take a lot of blood.”* (FGD 1)

Despite the pain, the overriding opinion was that any pain felt was quickly forgotten.

*“You feel pain and you forget it, it’s not like you stop coming for antenatal clinic just because of that.”* (FGD 5)

Many expressed a preference for the venous method as the same blood sample could be used for multiple RDTs.

*“I like my blood being taken from the arm because the blood taken is used to find so many diseases.”* (FGD 2)

##### Purpose and reliability of screening test results based on blood sampling method

There was some confusion between the belief that the test could detect malaria and the method of taking blood. For some there appeared to be an intrinsic link between the method of taking blood and the disease being tested.

*“Most of the time when they are using the finger prick tests it means they want to test the blood for HIV.”* (FGD 2)

*“I prefer peripheral tests to RDTs because when they do peripheral tests they test us for a lot of infections like syphilis, gonorrhoea, AIDS. They can diagnose us, unlike doing finger prick tests, they only test for one infection.”* (FGD 5)

Sentiments were expressed that venous blood samples provided more reliable results on malaria than finger-pick blood samples though there were also contrary opinions that the ability for a screening test to diagnose infection was not dependent on the method used to collect the blood.

*“When they are doing the finger prick test they don’t take a lot of blood, as a result sometimes it’s difficult for them to detect the parasite that causes malaria, unlike when they are taking the blood from the arm.”* (FGD 2)

*“If they use the finger prick test, they would say that I don’t have any disease when in actual fact, I have it. I don’t trust it.”* (FGD 2)

*“I think they can take blood from anywhere in the body and still test it, it’s the same blood.”* (FGD 2)

##### Health decision making empowerment

The act of taking a finger prick test was seen as empowering for pregnant women to be able to make informed decisions to ensure the health of themselves and their unborn babies. The benefits of blood tests were deemed to outweigh the discomfort experienced during the sampling procedure.

*“We know [after having the test] how we are in our body and if we are not okay, we are able to protect ourselves before the problems arises.”* (FGD 4)

##### Rumours regarding the purpose of blood samples

Participants reported social rumours regarding blood samples as their own opinions, the beliefs of their community or as both, and it was often difficult to differentiate between these viewpoints. As similarly reported by health workers, some FGD participants thought blood taken during the trial was being sold, with community perceptions that women enrolled in the trial were selling their blood for the value of the incentives provided.

*“Some said that they are taking your blood in exchange for soap or anything that you are given there and yet they are making profits out of your blood.”* (FGD 4)

Some FGD participants reported community perceptions that blood samples were kept or taken for occult practices. Another belief mentioned during the FGDs was that the failure by a woman to attend ANC visits would require repayment of a debt by giving blood.

*“Others were saying that if you didn’t go for antenatal clinic, they would follow you home so that you should repay the debt.”* (FGD 4)

However, there were no indications that these rumours would be sustained if similar volumes of blood were to be drawn as part of a screening policy in routine service provision which would not be accompanied by any incentives, or follow up visits at home.

By agreeing to have their blood tested, some FGD participants believed they were helping their friends who have a “shortage of blood”. Others said blood tests would lead to the birth of a small baby. Although such rumours emerged spontaneously in each FGD and were not prompted by the facilitator, in the discussions that followed many women said they did not believe the rumours and that they are wrong.

#### Drugs

The effectiveness and replacement of drugs to treat and prevent malaria, perceptions of prevention versus treatment, and opinions between DOT and continued unsupervised medication at home were sub-themes that emerged from the FGDs.

##### Drug effectiveness and replacement for prevention and treatment

Some women within the FGDs remained satisfied with SP, stating it still offered protection from malaria infection. However, other FGD participants believed SP no longer served its purpose and instead left them feeling weak, causing them to visit ANC again.

*“Sometimes when you took the Fansidar [SP] it wasn’t serving its purpose in the body and you could go again to be given another one.”* (FGD 1)

DP was commonly considered as “the new SP” and received positive views by the women, including that it gave women strength, was very effective and made malaria disappear. Women said DP tablets were slightly bigger and a different shape, but that they had no problems taking them.

*“Because when we were given this drug, we were protected and we never got sick again. Before I started antenatal clinic visits I used to get sick and when I came here and was given this new drug, I got some strength and I was able to work but I wouldn’t work beforehand.”* (FGD 1)

None of the women reported having experienced side effects with DP. In contrast, there were several reports that SP sometimes led to dizziness or nausea. This was especially associated with receiving SP as IPTp without a confirmed diagnosis of malaria.

*“Others when they were given Fansidar [SP], they were experiencing nausea, so when they were given this new drug they were able to differentiate because they did not experience any side effects.”* (FGD 4)

*“When we used to receive Fansidar [SP] without being tested…since you take the drug when not sick, you started feeling some odd things in the body.”* (FGD 1).

##### Prevention *versus* treatment

SP was strongly associated with prevention, providing relief for a short period of time rather than treatment, whereas DP was seen exclusively as treatment.

*“There is a difference between these two drugs; Fansidar [SP] protects one from malaria, but then after some time malaria symptoms resurfaces again while this new drug [DP] heals or rather cures so it’s good to go for the new drug because it cures.”* (FGD 2)

##### DOT versus continued medication at home

Only a few women in each FGD engaged with the issue of adherence, but those who did conceded it was sometimes difficult to take the full DP course. When asked about likely adherence to completing the full course of DP at home outside a trial setting, it was suggested that there may be laziness by some women to comply with required adherence and a risk that tablets may be misplaced by children in the home.

*“They give you the medication, you take it and because sometimes us women we may be lazy to take the medication, we may not properly take it [at home].”* (FGD 1)

Opinion was equally divided as to whether it would be better to take DP at the ANC or at home due to difficulty in daily travel or getting permission from their husbands. No opinions were expressed about adherence to SP as SP was routinely administered as DOT both in the trial and as experienced by the women in previous routine healthcare.

#### Reasons for repeat visits

Expectations and previous experiences at prior antenatal visits were major influences amongst pregnant women to attend subsequent antenatal care visits. Being well received and respectfully handled by health workers, informed about the wellbeing of their unborn baby and reassured of their own health by being tested for HIV and other infections, and receipt of medications and LLINs were expectations and experiences cited to have promoted high attendance of subsequent visits.

## Discussion

**Fig. 1:**
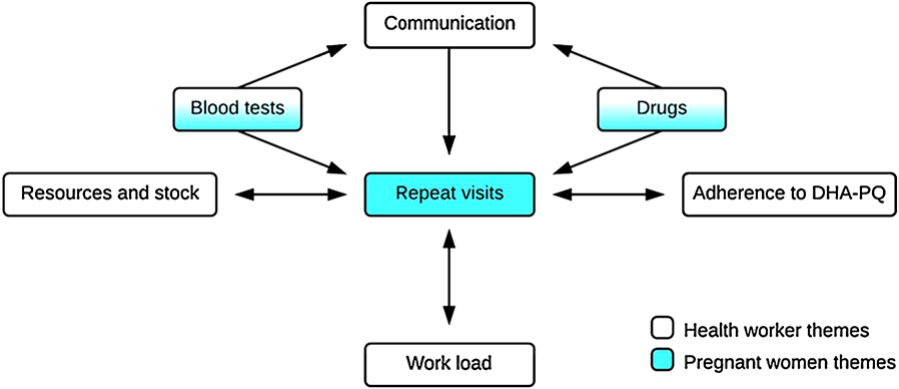
The relationship between opinions held by health workers and pregnant women of intermittent screening and treatment for malaria in pregnancy

Acceptance of ISTp by both health workers and pregnant women revolves around several aspects of the intervention as well as interactions between health workers and pregnant women and wider community perceptions. The study findings show that the opinions and perceptions of health workers and pregnant women involved in the ISTp trial were interlinked and overlapped on several aspects (Fig. 1). Some of the factors underlying acceptance of ISTp-DP as an alternative strategy to IPTp-SP were specific to the particular elements of ISTp-DP. Other acceptability issues link more to feasibility of effective implementation of the strategy under operational conditions and how this could influence motivation to attend ANC, and so are affected by broader health system constraints.

Repeat visits of women to the ANC during their pregnancy is central to the success of ISTp both within the trial and if IST were to become a policy. As seen elsewhere [27–30], this study found that pregnant women do not attend ANC solely for malaria screening; they want the progress of their babies’ development to be monitored to increase the chance that the baby will be born healthy and without complications. Furthermore, the study findings strongly suggest that the likelihood of pregnant women complying with repeat visits is related to the quality of services and care that they received. All women reported their satisfaction with receiving testing for infections which are provided as part of routine ANC (HIV, syphilis and gonorrhoea) and those in the ISTp arm were happy to attend repeat ANC visits to receive testing for malaria. It is important to note however, that additional resources were available to women enrolled in the trial for transportation and other incentives, such as LLINs and soap, which would not be available under operational conditions. In the context of the trial, health workers reported focusing on the increased availability of resources and stock within the study to encourage participation and repeat visits. This indicates that resources and stock may have been identified by pregnant women as an obstacle to having their expectations met and underlines the importance of monitoring to ensure it does not have a future impact on acceptability of ANC services once the trial is finished.

An intervention requiring greater implementation input will lead to an increase in work load for service providers. Although some health workers stated their work load was manageable, others did not agree, and expressed concern that it would worsen with the implementation of ISTp as there was “more to be done”. Reasons for these mixed opinions may reflect uneven work distribution between the different cadres of staff interviewed, or differing perceptions of work load. Interestingly, however, some health workers predicted that their work load might actually decrease if ISTp-DP replaced IPTp-SP due to fewer unscheduled visits by women attending with recurrent clinical malaria in light of perceived reduced prophylactic efficacy of IPTp-SP.

Common sub-themes regarding blood tests were discussed by health workers and pregnant women. For example, all were generally supportive of the need and importance of blood tests to test for infections during pregnancy, and that although taking blood causes discomfort this is outweighed by the benefits to mother and baby. Many pregnant women agreed with the idea of being tested for an infection before receiving treatment citing the discomfort of side effects of unnecessary drugs. This is in line with findings of other studies exploring the acceptability of ISTp in Ghana and Kenya [27, 28, 30]. The importance of clear communication in relation to what the tests are for, and their results was emphasized by both pregnant women and health workers. For example, women reported that being able to see the result of an RDT in front of them was a very positive factor in believing the diagnosis. Nevertheless, there were contrasting opinions amongst health workers and pregnant women about whether microscopy or an RDT was more accurate for diagnosing malaria, and likewise with respect to whether results were more accurate for blood taken venously or by finger prick.

Although RDTs were described as simple and accurate by most health workers, not all were in agreement over RDT accuracy, as supported by evidence from elsewhere on non-adherence to negative RDTs in the diagnosis and treatment of clinical malaria [31–34]. Evidence on RDT use amongst pregnant women is limited [35] but a recent multi-site qualitative study of the prevention and treatment of malaria in pregnancy suggests that trust in RDT results is influenced by the complex relationship between biomedical malaria and its overlap with general symptoms and signs of pregnancy. Generally, positive RDTs were seen as confirmation of malaria whereas negative RDTs were not trusted as confirmation of lack of malaria, as the parasites could be “hiding” and this could lead to mistrust of the accuracy of RDTs [29, 36]. Comments from pregnant women and health facility staff in the study also seem to support this observation. This is important given the results of the current trial which found that RDTs only detected around 45% of PCR-positive infections in paucigravidae and 30% in multigravidae; as women were treated according to RDT result, this may explain the higher prevalence of malaria infections at delivery in the ISTp-DP arm [22]. However, it is important to note that the biological impact of such low-level parasitaemia during pregnancy is unclear [37, 38] and the incidence of clinical malaria was not significantly different between the trial arms [22]. Compliance with RDT result was not an issue in the trial setting but it is unclear whether or not non-compliance would be observed in a screening strategy involving asymptomatic pregnant women under routine conditions. Compliance with best practices for the operation of RDTs, and an appreciation of the sensitivity and specificity of RDTs by health workers would need to be emphasized in the context of a screening strategy to increase health worker acceptability [39] and efficiency of implementation of the strategy.

Health staff and pregnant women were generally happy with the communication between them. This is in contrast to other studies which found that poor communication or staff rudeness contributed to poor ANC attendance [40]. This difference may be a reflection of the trial setting where clear communication is crucial to address concerns and encourage high enrolment and retention. Health worker opinions of blood tests and their perceptions of how pregnant women feel about blood tests are likely to affect how they communicate their purpose and importance which could be a possible impeding or facilitating factor within the routine setting to the acceptability of ISTp.

According to reports from the health workers and women in this study, rumours and misconceptions were prevalent within the communities regarding venous blood draws for screening purposes. Although the sentiments of the rumours did not seem to be shared by health workers or trial participants, the adverse consequences of the community rumours could prove to be a barrier to women attending antenatal care if IST were to be implemented as policy, being integrated with other routine screening tests such as HIV, syphilis and anaemia. This could potentially be addressed by the use of finger prick as opposed to venous blood draws in routine practice, providing a transparent and accountable mechanism regarding use of blood samples. The presence of more unusual and damaging rumours underlines the importance of well-trained local health workers that are able to address them appropriately without generating further fear. The general feeling among health workers was that any misunderstanding can be rectified if it is properly explained and that health education should be emphasized within the community, as supported by a similar study assessing acceptability of ISTp in Ghana [41].

The opinions of both groups of respondents on SP and DP were also interlinked. A key challenge is separating opinions of the components of the ISTp intervention with the drug used as the two are inextricably linked. Both health workers and pregnant women reported observing that women in the IPTp-SP arm of the trial were frequently ill and experienced more of the consequences of malaria in pregnancy and many attributed this to the reduced efficacy of SP. DP was the component of ISTp most strongly approved of by health workers in light of the perceived inferiority of SP. Both health workers and pregnant women also perceived that DP had fewer side effects than SP for women enrolled in the trial. This is interesting, given that the trial found no significant differences in clinical malaria or adverse events between the two arms [22]. Sentiments regarding the effectiveness of either drug by both health workers and pregnant women influenced their opinions of the concept of prevention versus cure. This was not thought of great importance by either due to the overall feeling that IPTp-SP no longer provided sufficient protection; a greater preference was placed instead on an approach that would combine prevention and effective treatment instead of differentiating between them: prevent first (with more emphasis being placed on other preventive measures such as ITNs) and then treat if the prevention has not worked and a woman is found to be positive for malaria parasites. Interestingly, this suggestion of a hybrid approach also arose from the ISTp-DP acceptability study in Kenya [28].

The participants of this Malawi acceptability study had not had any prior exposure to DP for the treatment or prevention of malaria prior to enrolment in the trial as it is not registered or available in the public sector in Malawi. DP for the treatment of confirmed malaria is a 3-day course of multiple tablets taken once daily as opposed to a single dose of SP taken for IPTp under the supervision of a health worker. This raised concerns by health workers about adherence to the full course if ISTp-DP were to be introduced under operational conditions. FGD participants in the ISTp arm that had been diagnosed with malaria during their pregnancy were pleased with the DP they received, but most did not engage with the issue of adherence, which was a concern raised more by the health workers interviewed. This was possibly due to the trial setup as DP was taken under clinical supervision and if the pregnant women did not attend ANC for their dose they were contacted by a member of the study team who provided transport if necessary. A woman’s experience of ANC does not begin in the consultation room but with the journey to the health facility, which may be a considerable distance. Health workers were concerned that women may not take all doses of DP if unsupervised, but likewise that transport limitations may be a significant barrier to pregnant women’s acceptability of ISTp if DOT of all three doses at a health facility was the recommendation. Previous research in non-pregnant populations has indicated moderate adherence to artemether–lumefantrine (AL) for uncomplicated malaria in rural Malawi [42, 43] but good unsupervised self-dosing of AL in Tanzania [44] and of artesunate–amodiaquine in Ghana [23]. The evidence base for adherence to treatment of malaria with an ACT during pregnancy is smaller and mixed. For example, a recent study from the Gambia [45] reported that despite high knowledge of the biomedical importance of malaria infection by pregnant women, adherence to anti-malarial treatment was low, usually discontinuing treatment after prescription for several reasons including perceived ineffectiveness of treatment, risk of medication, family influence, and denial of diagnosis of disease. In contrast, a study of the acceptability of ISTp alongside a clinical trial in Northern Ghana found that adherence to self-administered AL by trial participants (determined by inspection of blister packets) was high [30]. Helpful packaging, pictorial dosing instructions and patient conviction that the strategy is effective can all contribute to increased adherence [44] although barriers to compliance with self-continued treatment of ISTp-DP by pregnant women at home would require careful attention.

## Limitations

Although the findings of the study favour the acceptability of ISTp-DP as an alternative to IPTp-SP, it is important to recognize that the experiences and opinions of the two strategies expressed by pregnant women and health providers in this study were within the context of a clinical trial. The additional resources that this brings, including uninterrupted supplies of RDTs and anti-malarials, trial staff dedicated to the delivery of the interventions, direct observation of all doses of DP and reimbursement of travel expenses for participants, are unlikely to be replicated if ISTp-DP were to be implemented under routine care. Similarly, the strong levels of communication between health workers, trial staff and participants may have influenced the attitudes and perceptions of the pregnant women with respect to the efficacy and acceptability of IPTp-SP and IST-DP. The perceived superiority of DP compared to SP for example is in contrast to the trial results which found no significant difference in clinical malaria between the two arms and may be a result of respondents believing that the new drug must be better than the existing one it was being tested against; due to the nature of the interventions, study participants and health staff could not be blinded to the arm of allocation.

In addition, recruitment of pregnant women to the FGDs depended on who was attending the clinic for their postpartum follow up visit on the day that the study team was present. As such, it was not possible to design the composition of the FGDs with respect to trial arm and experience of the different interventions as has been done in previous studies [27, 28]. This means that the women involved in the qualitative study may not be fully representative of the trial population as a whole. However, women of different age, parity and trial arm were included and a range of opinion was elicited. Nevertheless, it is possible that the small number of FGDs means that saturation of opinions may not have been reached.

It would be of interest to conduct a feasibility study in this setting to explore the relative importance of these factors that would influence effective implementation of ISTp-DP if it were to become policy.

## Conclusion

This is the first study to explore health worker and pregnant women acceptability of ISTp within a trial context in Malawi and to explore the possible implications for acceptability were ISTp to become national policy. In order for ISTp with DP to be an acceptable strategy, pregnant women must understand the consequences of any malaria infection during pregnancy, the importance of scheduled screening for malaria and, if diagnosed with malaria, adherence to treatment in an unsupervised setting. This understanding must be guided by a clear message from health workers to avoid the emergence of rumours and misconception. Although IPTp was acknowledged by some health workers to have advantages over ISTp, such as simplicity and compliance, almost all focused on its drawbacks particularly a perceived decrease in effectiveness with increasing SP resistance. Overall, they approved of ISTp and wanted it to become a permanent policy in place of IPTp. There was strong agreement that pregnant women would accept and adhere to ISTp if it were to become government health policy as it is viewed as compulsory; this is supported by pregnant women’s trust in services provided during their antenatal care which will improve the health of their babies. The interviews and FGDs analysed in this paper were conducted before the results of the trial were published and IST-DP was found to be non-superior to IPTp-SP in this setting. However, the factors influencing acceptability of ISTp cannot be separated from factors influencing acceptability of focused ANC, for example an appreciation by pregnant women of regular blood tests and strong communication about the progress of their pregnancy. Adherence to scheduled ANC visits along with the best malaria control measures available should be further encouraged with emphasis placed on community-based strategies to strengthen ties between the formal health sector and local communities. However, feasibility factors that would influence quality of services provided such as a reliable supply of commodities must also be considered in determining the potential success of any strategy for the control of malaria in pregnancy.

## References

[1] Desai M, ter Kuile FO, Nosten F, McGready R, Asamoa K, Brabin B (2007). Epidemiology and burden of malaria in pregnancy. Lancet Infect Dis.

[2] Cornet M, Le Hesran JY, Fievet N, Cot M, Personne P, Gounoue R (1998). Prevalence of and risk factors for anemia in young children in southern Cameroon. Am J Trop Med Hyg.

[3] van Eijk AM, Ayisi JG, ter Kuile FO, Misore AO, Otieno JA, Kolczak MS (2002). Malaria and human immunodeficiency virus infection as risk factors for anemia in infants in Kisumu, western Kenya. Am J Trop Med Hyg.

[4] Reed SC, Wirima JJ, Steketee RW (1994). Risk factors for anemia in young children in rural Malawi. Am J Trop Med Hyg.

[5] Le Hesran JY, Cot M, Personne P, Fievet N, Dubois B, Beyeme M (1997). Maternal placental infection with *Plasmodium falciparum* and malaria morbidity during the first 2 years of life. Am J Epidemiol.

[6] Malhotra I, Dent A, Mungai P, Wamachi A, Ouma JH, Narum DL (2009). Can prenatal malaria exposure produce an immune tolerant phenotype? A prospective birth cohort study in Kenya. PLoS Med.

[7] Mutabingwa TK, Bolla MC, Li JL, Domingo GJ, Li X, Fried M (2005). Maternal malaria and gravidity interact to modify infant susceptibility to malaria. PLoS Med.

[8] Schwarz NG, Adegnika AA, Breitling LP, Gabor J, Agnandji ST, Newman RD (2008). Placental malaria increases malaria risk in the first 30 months of life. Clin Infect Dis.

[9] O’Neil-Dunne I, Achur RN, Agbor-Enoh ST, Valiyaveettil M, Naik RS, Ockenhouse CF (2001). Gravidity-dependent production of antibodies that inhibit binding of *Plasmodium falciparum*-infected erythrocytes to placental chondroitin sulfate proteoglycan during pregnancy. Infect Immun.

[10] Slutsker L, Marston BJ (2007). HIV and malaria: interactions and implications. Curr Opin Infect Dis.

[11] Anders K, Marchant T, Chambo P, Mapunda P, Reyburn H (2008). Timing of intermittent preventive treatment for malaria during pregnancy and the implications of current policy on early uptake in north-east Tanzania. Malar J.

[12] Mubyazi GM, Magnussen P, Goodman C, Bygbjerg IC, Kitua AY, Olsen OE (2008). Implementing intermittent preventive treatment for malaria in pregnancy: review of prospects, achievements, challenges and agenda for research. Open Trop Med J.

[13] Mbonye AK, Magnussen P (2010). Symptom-based diagnosis of malaria and its implication on antimalarial drug use in pregnancy in Central Uganda: results from a community trial. Int J Adolesc Med Health.

[14] WHO (2004). A strategic framework for malaria prevention and control during pregnancy in the African Region.

[15] WHO (2014). Policy brief for the implementation of intermittent preventive treatment of malaria in pregnancy using sulfadoxine–pyrimethamine (IPTp-SP).

[16] Peters PJ, Thigpen MC, Parise ME, Newman RD (2007). Safety and toxicity of sulfadoxine/pyrimethamine: implications for malaria prevention in pregnancy using intermittent preventive treatment. Drug Saf.

[17] Bardaji A, Bassat Q, Alonso PL, Menendez C (2012). Intermittent preventive treatment of malaria in pregnant women and infants: making best use of the available evidence. Expert Opin Pharmacother.

[18] ter Kuile FO, van Eijk AM, Filler SJ (2007). Effect of sulfadoxine–pyrimethamine resistance on the efficacy of intermittent preventive therapy for malaria control during pregnancy: a systematic review. JAMA.

[19] Feng G, Simpson JA, Chaluluka E, Molyneux ME, Rogerson SJ (2010). Decreasing burden of malaria in pregnancy in Malawian women and its relationship to use of intermittent preventive therapy or bed nets. PLoS ONE.

[20] Nkhoma S, Molyneux M, Ward S (2007). Molecular surveillance for drug-resistant *Plasmodium falciparum* malaria in Malawi. Acta Trop.

[21] Villar BJ (2003). WHO antenatal care randomized trial; manual for the implementation of the new model.

[22] Madanitsa M, Kalilani L, Mwapasa V, van Eijk AM, Khairallah C, Ali D (2016). Scheduled intermittent screening with rapid diagnostic tests and treatment with dihydroartemisinin–piperaquine versus intermittent preventive therapy with sulfadoxine–pyrimethamine for malaria in pregnancy in Malawi: an open-label randomized controlled trial. PLoS Med.

[23] Tagbor H, Bruce J, Agbo M, Greenwood B, Chandramohan D (2010). Intermittent screening and treatment versus intermittent preventive treatment of malaria in pregnancy: a randomised controlled non-inferiority trial. PLoS ONE.

[24] National Malaria Control Programme (NMCP) and ICF International (2014). Malawi malaria indicator survey (MIS).

[25] Kalilani-Phiri L, Thesing PC, Nyirenda OM, Mawindo P, Madanitsa M, Membe G (2013). Timing of malaria infection during pregnancy has characteristic maternal, infant and placental outcomes. PLoS ONE.

[26] Bernard HR (2006). Analysis of qualitative data. Research methods in anthropology: qualitative and quantitative approaches.

[27] Smith LA, Jones C, Adjei RO, Antwi GD, Afrah NA, Greenwood B (2010). Intermittent screening and treatment versus intermittent preventive treatment of malaria in pregnancy: user acceptability. Malar J.

[28] Hill J, Hoyt J, Achieng F, Ouma P, L’Lanziva A, Kariuki S (2016). User and provider acceptability of intermittent screening and treatment and intermittent preventive treatment with dihydroartemisinin–piperaquine to prevent malaria in pregnancy in Western Kenya. PLoS ONE.

[29] Pell C, Menaca A, Afrah NA, Manda-Taylor L, Chatio S, Were F (2013). Prevention and management of malaria during pregnancy: findings from a comparative qualitative study in Ghana, Kenya and Malawi. Malar J.

[30] Pell C, Menaca A, Chatio S, Hodgson A, Tagbor H, Pool R (2014). The acceptability of intermittent screening and treatment versus intermittent preventive treatment during pregnancy: results from a qualitative study in Northern Ghana. Malar J.

[31] Barat L, Chipipa J, Kolczak M, Sukwa T (1999). Does the availability of blood slide microscopy for malaria at health centers improve the management of persons with fever in Zambia?. Am J Trop Med Hyg.

[32] Reyburn H, Mbatia R, Drakeley C, Carneiro I, Mwakasungula E, Mwerinde O (2004). Overdiagnosis of malaria in patients with severe febrile illness in Tanzania: a prospective study. BMJ.

[33] Asiimwe C, Kyabayinze DJ, Kyalisiima Z, Nabakooza J, Bajabaite M, Counihan H (2012). Early experiences on the feasibility, acceptability, and use of malaria rapid diagnostic tests at peripheral health centres in Uganda-insights into some barriers and facilitators. Implement Sci.

[34] Chandler CI, Mangham L, Njei AN, Achonduh O, Mbacham WF, Wiseman V (2012). As a clinician, you are not managing lab results, you are managing the patient: how the enactment of malaria at health facilities in Cameroon compares with new WHO guidelines for the use of malaria tests. Soc Sci Med.

[35] Hill J, D’Mello-Guyett L, Hoyt J, van Eijk AM, ter Kuile FO, Webster J (2014). Women’s access and provider practices for the case management of malaria during pregnancy: a systematic review and meta-analysis. PLoS Med.

[36] Menaca A, Pell C, Manda-Taylor L, Chatio S, Afrah NA, Were F (2013). Local illness concepts and their relevance for the prevention and control of malaria during pregnancy in Ghana, Kenya and Malawi: findings from a comparative qualitative study. Malar J.

[37] Williams JE, Cairns M, Njie F, Laryea Quaye S, Awine T, Oduro A (2016). The performance of a rapid diagnostic test in detecting malaria infection in pregnant women and the impact of missed infections. Clin Infect Dis.

[38] Cottrell G, Moussiliou A, Luty AJ, Cot M, Fievet N, Massougbodji A (2015). Submicroscopic *Plasmodium falciparum* infections are associated with maternal anemia, premature births, and low birth weight. Clin Infect Dis.

[39] Mbacham WF, Mangham-Jefferies L, Cundill B, Achonduh OA, Chandler CIR, Ambebila JN (2014). Basic or enhanced clinician training to improve adherence to malaria treatment guidelines: a cluster-randomised trial in two areas of Cameroon. Lancet Glob Health.

[40] Mannava P, Durrant K, Fisher J, Chersich M, Luchters S (2015). Attitudes and behaviours of maternal health care providers in interactions with clients: a systematic review. Glob Health.

[41] Smith Paintain L, Antwi GD, Jones C, Amoako E, Adjei RO, Afrah NA (2011). Intermittent screening and treatment versus intermittent preventive treatment of malaria in pregnancy: provider knowledge and acceptability. PLoS ONE.

[42] Mace KE, Mwandama D, Jafali J, Luka M, Filler SJ, Sande J (2011). Adherence to treatment with artemether–lumefantrine for uncomplicated malaria in rural Malawi. Clin Infect Dis.

[43] Bell DJ, Wootton D, Mukaka M, Montgomery J, Kayange N, Chimpeni P (2009). Measurement of adherence, drug concentrations and the effectiveness of artemether–lumefantrine, chlorproguanil–dapsone or sulphadoxine–pyrimethamine in the treatment of uncomplicated malaria in Malawi. Malar J.

[44] Kabanywanyi AM, Lengeler C, Kasim P, King’eng’ena S, Schlienger R, Mulure N (2010). Adherence to and acceptability of artemether–lumefantrine as first-line anti-malarial treatment: evidence from a rural community in Tanzania. Malar J.

[45] Jaiteh F, Dierickx S, Gryseels C, O’Neill S, D’Alessandro U, Scott S (2016). ‘Some anti-malarials are too strong for your body, they will harm you’. Socio-cultural factors influencing pregnant women’s adherence to anti-malarial treatment in rural Gambia. Malar J.

